# A New Xanthone from the Bark Extract of *Rheedia acuminata* and Antiplasmodial Activity of Its Major Compounds

**DOI:** 10.3390/molecules15107106

**Published:** 2010-10-14

**Authors:** Guillaume Marti, Véronique Eparvier, Marc Litaudon, Philippe Grellier, Françoise Guéritte

**Affiliations:** 1 Centre de Recherche de Gif, Institut de Chimie des Substances Naturelles, CNRS, 1 avenue de la Terrasse, 91198 Gif-sur-Yvette Cedex, France; E-Mails: guillaume.marti@icsn.cnrs-gif.fr (G.M.); marc.litaudon@icsn.cnrs-gif.fr (M.L.); francoise.gueritte@icsn.cnrs-gif.fr (F.G.); 2 UPS2561, CNRS, 2, avenue Gustave Charlery, 97300 Cayenne, France; 3 Muséum National d’Histoire Naturelle, FRE 3206 CNRS, CP52, 61 rue Buffon, 75231 Paris cedex 05, France; E-Mail: grellier@mnhn.fr (P.G.)

**Keywords:** *Rheedia acuminata*, Clusiaceae, xanthones, antiplasmodial activity, cytotoxicity

## Abstract

Bioassay-guided fractionation of the ethyl acetate bark extract of *Rheedia acuminata* led to the isolation of the new compound 1,5,6-trihydroxy-3-methoxy-7-geranyl-xanthone (**1**), together with four known compounds **2**-**5**. These compounds were tested *in vitro* for their antiplasmodial activity on a chloroquine-resistant strain of *Plasmodium falciparum* (FcB1) and for their cytotoxicity against the human diploid embryonic lung cell line MRC-5.

## 1. Introduction

In South America several species of Clusiaceae are widely used in the manufacture of hulls, and well known for quality of their wood and for the healing properties of their latex used traditionally used for their effectiveness against dermatoses [1]. One of these, *Rheedia acuminata* (Ruiz & Pavon) Planchon and Triana, a tree growing in Amazonian rainforest, possesses an abundant latex used for various medicinal purposes by the Guianese Amerindians (Palikur) in the form of patches or breakdowns applied to the wrinkling muscle. In addition, *Rheedia acuminata* fruits are commonly consumed in South America [1]. 

Prenylated xanthones and **p**oly**p**renylated **a**cyl**p**hloroglucinols (PPAPs) are widely distributed in the Clusiaceae, the genus *Rheedia* being a rich source of them [2,3,4,5]. Some biflavonoids were also previously isolated from this species [6]. Their biological activities include antibacterial, analgesic and cytotoxic properties [5,7,8]. 

In an effort to find new natural antimalarial drugs and as part of investigation of French Guiana plants, we found that the ethyl acetate extract of the bark of *Rheedia acuminata* showed antiplasmodial activity (92% of inhibitory growth of *Plasmodium falciparum *(FcB1) at 10 µg/mL), whereas leaves and fruits extracts showed no significant and weak activity respectively (28% and 50% of inhibitory growth at 10 µg/mL, respectively). We report here the bioassay-guided fractionation of this extract on the basis of this antiplasmodial activity. 

## 2. Results and Discussion

**Figure 1:**
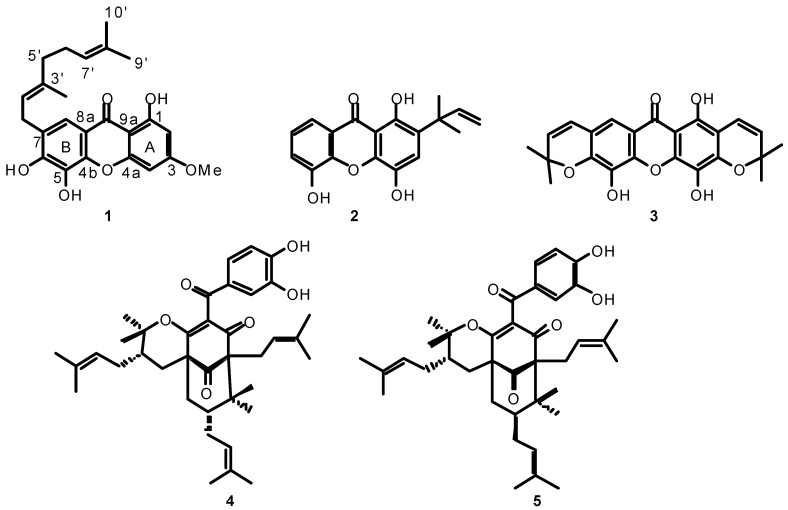
Structures of compounds **1**-**5**.

The bioguided fractionation of the ethyl acetate extract of trunk bark of *Rheedia acuminata* led to the isolation of the new 1,5,6-trihydroxy-3-methoxy-7-geranyl-xanthone (**1**) along with two xanthone analogues (**2** and **3**) and two PPAPs (**4 **and **5**) (Figure 1). 

**Figure 2:**
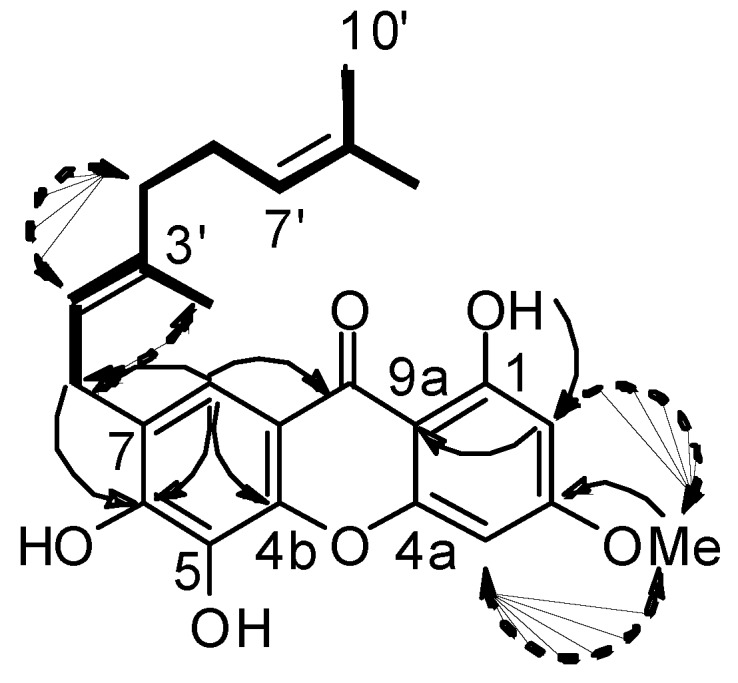
Key COSY (bold), HMBC (plain arrows) and NOESY (dashed arrows) correlations for **1**.

Compound **1** was obtained as a yellow oil. The HREIMS indicated a [M+H]^-^ ion peak at *m/z *409.1652, giving the molecular formula C_24_H_25_O_6_ (calc. 409.1651). The IR spectrum displayed free hydroxyl (3,410 cm^−1^), chelated hydroxyl (3,170 cm^-1^), conjugated carbonyl (1,640 cm^−1^) and aromatic ring (1,570 cm^−1^) peaks. These data, together with absorption bands observed at λ_max_ 251, 283 and 327 nm in the UV spectrum and those obtained from 1D and 2D NMR experiments of compound **1** suggested the presence of a 1,3,5,6-tetraoxygenated xanthone system [10,11]. Examination of the ^1^H- NMR spectrum showed the presence of three hydroxyl groups with two broad signals at δ_H_ 7.88, 12.68 and one singlet at δ_H_ 14.02 ppm, corresponding to a chelated proton, one singlet at δ_H_ 8.08, two *meta*-coupled protons at δ_H_ 6.56 and 6.04 (1H, d, *J* = 2.3 Hz), and one methoxyl (δ_H_ 3.63). In addition two vinyl protons at δ_H_ 5.77 and 5.26 (1H, *brt*, *J* = 6.8 Hz), three methylene groups at δ_H_ 3.83 (2H, *d*, *J* = 7.3 Hz), 2.21 (2H, *m*) and 2.15 (2H, *m*), and three methyl groups at δ_H_ 1.84, 1.69, 1.59 (3H, *s*) suggested the presence of a geranyl side chain. HSQC experiments allowed the assignment of all protonated carbons. A combination of HMBC, COSY and NOESY experiments were used to establish the position of the substituents. In the HMBC spectrum (Figure 2), the chelated proton OH-1 showed correlations with C-2 at δ_c_ 97.6 on one hand, and the proton H-2 with carbons C-9a (δ_C_ 104.2), C-4 (δ_C_ 92.8), C-1 (δ_C_ 164.7) and C-3 (δ_C_ 166.7) on the other hand. A methoxy group was deduced from the correlation between the methyl protons at δ_H_ 3.63 with C-3. NOESY correlations between the methoxy at C-3 with protons H-2 (δ_H_ 6.56) and H-4 (δ_H_ 6.04) confirmed the position of the substituents on ring A (Figure 2). Furthermore, HMBC correlations observed between H-8 and C-9 (δ_C_ 181.5) were indicative of a *peri* location of the carbonyl. In addition, proton H-8 showed correlations with C-6 (δ_C_ 153.4), C-4b (*δ*_C_ 146.6), and CH_2_-1’ (*δ*_C_ 29.5) and a long-range correlation with C9a (δ_C_ 104.2). The geranyl side chain was deduced from the COSY spectrum with, on one hand, correlations observed between Me-9’ and Me-10’ [δ_H_ 1.59 (3H, *s*, H-9’) and 1.69 (3H, *s*, H-10’)] and the vinylic proton H-7’ at δ_H_ 5.28 (1H, *brt*, *J *= 6.8 Hz), which in turn showed a cross peak with H_2_-6’(δ_H_ 2.21, 2H, *m*). On the other hand, correlations observed between H_2_-5’ (δ_H_ 2.16, 2H, *m*) and H_2_-6’and H-2’ at δ_H_ 5.77 (1H, *brt*, *J *= 6.8 Hz), which in turn showed correlations with Me-4’(δ_H_ 1.84, 3H, *s*) and H_2_-1’(δ_H_ 3.83, 2H, *d*, *J* = 7.3 Hz) confirmed the presence of a geranyl side chain. The *E* configuration of the C-2’-C-3’ double bond was established by NOESY correlations observed between H_2_-5’ with H-2’, and Me-4’with H_2_-1’ (Figure 2). Finally, the position of the geranyl side chain at C-7 (δ_C_ 127.8) was confirmed by HMBC correlation from H_2_-1’ to C-6 (δ_c_ 153.4) (Figure 2). Compound **1**, which was named 1,5,6-trihydroxy-3-methoxy-7-geranylxanthone, is a positional isomer of cowaxanthone and rubraxanthone isolated from *Garcinia cowa * [12,13].

Compound **2 **was identified as 2-(1’-1’-dimethylprop-2’-enyl)-1,4,5-trihydroxyxanthone and compound **3** as pyrojacareubin by comparison of the UV, HRESIMS, 1D and 2D NMR with the literature data [13,14,15]. The 1D and 2D NMR spectra associated with their optical rotation values confirmed that **4** was isogarcinol [16,17,18] and **5**, 7-*epi*-isogarcinol [16]. 

The antiplasmodial activity against the chloroquine-resistant strain of *P. falciparum* FcB1 and the cytotoxicity upon the human diploid embryonic cell line MRC-5 of compounds **1-5** are summarized in Table 1. The three xanthones **1**, **2** and **3** isolated from *Rheedia acuminata* bark have IC_50_s against *Plasmodium falciparum* exceeding 10 µM. Compounds **4** and **5** have shown the best activity with IC_50_s around 3 µM. The IC_50_ values obtained on MRC-5 cell line indicated that PPAPs **4** and **5** exhibited significant cytotoxicity whereas xanthones (**1** to **3**) showed weak cytotoxicity. Several xanthones, which have been isolated from various species of the family Clusiaceae, showed cytotoxic and antiplasmodial activities [9,10,19,20] and structure-activity relationships have been proposed in this series. For example, Winter *et al.* have demonstrated that xanthones with a hydroxyl group in *peri* positions with regards to the carbonyl (such as **1**, **2** and **3)**, possess weak antiplasmodial activity due to their low affinity for the heme [21,22]. In conclusion, the antiplasmodial activity detected in the crude extract is probably due to the presence of high quantity of the two PPAPs **4** and **5**.

**Table 1:** IC_50_ values of compounds **1**-**5** tested against FcB1 strain of *P. falciparum* and MRC-5 cell.

Compound	IC_50_ *P.f.* FcB1 (µM) ± SD (n=3)	IC_50_ MRC-5 (µM) ± SD (n=3)
**1**	10.5^a^	36^a^
**2**	15.1^a^	b
**3**	11.4^a^	29^a^
**4**	3.5 ± 1.1	3.5 ± 0.4
**5**	3.2 ± 1.3	2.3 ± 0.5
Chloroquine	0.078 ± 0.006	
Taxotere		31.5 ± 4.5

^a^ biological assays in duplicates; ^b ^not enough material.

## 3. Experimental

### 3.1. General

The NMR spectra were recorded on a Bruker 500 MHz (Avance 500) or 300 MHz (Aspect DPX 300 MHz) spectrometer. ESIMS were obtained on a Thermoquest Navigator mass spectrometer. HRESIMS were obtained on a ESI-TOF spectrometer (LCT; Waters). Kromasil analytical, semi-preparative and preparative C-18 columns (250 × 4.5 mm; 250 × 10 mm and 250 × 21.2 mm I.D, 5 µM Thermo^®^) were used for preparative HPLC separations using a “Waters autopurification system” equipped with a sample manager (Waters 2767), a column fluidics organizer, a binary pump (Waters 2525), a UV-Vis diode array detector (190-600nm), Waters 2996) and PL-ELS 1000 ELSD detector Polymer laboratory. IR spectra were obtained on a Nicolet FTIR 205 spectrophotometer. The UV spectra were recorded on a Perkin-Elmer Lambda 5 spectrophotometer. Specific rotation was obtained in CHCl_3_ with a JASCO P-1010 polarimeter. Silica gel 60 (35–70 µm) and analytical TLC plates (Si gel 60 F 254) were purchased from SDS (France). Polyamide DC 6 and polyamide cartridge were purchased from Macherey-Nagel (Chromabond PA, 1 g). 

### 3.2. Plant Material

Trunk bark of *R. acuminata* were collected in French Guiana. A voucher specimen (PMF-258) was deposited in the Herbarium of Cayenne (French Guiana), and identified by M-F. Prévost (IRD). 

### 3.3. Extraction and Isolation

Trunk bark (370 g) were extracted three times with EtOAc (3 × 1L) at 40 °C and 1,450 psi on a Zippertex^®^ static high-pressure high-temperature extractor developed in the ICSN Pilot Unit. This extract (10 g) was filtered on polyamide. Filtered extract (9.3 g) was subjected to silica gel chromatography using heptane/EtOAc mixtures (100/0-0/100). According to their TLC profile, 10 fractions were obtained (F1-F10). Fraction F4 (1.1 g), which showed antiplasmodial activity, was subjected to a preparative C-18 column using an isocratic mobile phase consisting of ACN/water + 0.1 % formic acid (flow rate 21 mL/min). This resulted in the isolation of **4** (72 mg, 0.019%), **5** (65 mg, 0.017%), **3** (2 mg, 0.0008%), **1** (8 mg, 0.0021%) and **2** (18 mg, 0.004%) with retention times of 15.5, 13.5, 10.4, 9.4, and 4.6 mins, respectively.

*1,5,6-Trihydroxy-3-methoxy-7-geranylxanthone (**1**):* Yellow oil; UV (MeOH) λ_max_ (log ε): 327 (3.99), 283 (3.96), 251 (4.35); IR ν_max_ (ns) 3410, 1640, 1570, 1450, 1370, 1290, 1220 cm^-1^; ^1^H-NMR (pyridine-*d*_5_, 500 MHz): δ 14.02 (1H, *s*, OH-1), 12.68 (1H, *brs*, OH), 8.08 (1H, *s*, H-8), 7.88 (1H, *brs*, OH) 6.56 (1H, *d*, *J *= 2.3 Hz, H-2), 6.04 (1H, *d*, *J *= 2.3 Hz, H-4), 5.77 (1H, *brt*, *J *= 6.8 Hz, H-2’), 5.28 (1H, *brt*, *J *= 6.8 Hz, H-7’), 3.83 ( 2H, *brd*, *J *= 7.3 Hz, H-1’), 3.63 (3H, *s*, OMe-3), 2.21 (2H, *m*, H-6’), 2.15 (2H, *m*, H-5’), 1.84 (3H, *s*, H-4’), 1.69 (3H, *s*, H-10’), 1.59 (3H, *s*, H-9’); ^13^C-NMR (pyridine-*d*_5_, 125 MHz): δ 181.5 (C-9), 166.7 (C-3), 164.7 (C-1), 158.5 (C-4a), 153.4 (C-6), 146.6 (C-4b), 137.2 (C-3’), 133.9 (C-5), 131.8 (C-8’), 127.8 (C-7), 125.3 (C-7’), 123.5 (C-2’), 116.7 (C-8), 113.8 (C-8a), 104.2 (C-9a), 97.6 (C-2), 92.8 (C-4), 56.2 (O-CH_3_), 40.5 (C-5’), 29.5 (C-1’), 27.5 (C-6’), 26.2 (C-9’), 17.7 (C-4’), 18.2 (C-10’); HREIMS [M-H]^-^*m/z* 409.1697, C_24_H_26_O_6_ requires 409.1651. 

*2-(1’,1’-Dimethylprop-2’-enyl)-1,4,5-Trihydroxyxanthone* (**2**): Yellow powder; UV (MeOH) λ_max_ (log ε): 320 (3.61), 263 (4.12), 249 (4.12), 236 (4.07); IR ν_max_ (ns) 3570, 1720, 1680, 1540, 1270, 1120 cm^-1^; ^1^H-NMR (pyridine-*d*_5_, 500 MHz): δ 13.55 (1H, *s*, -OH), 7.99 (1H, *dd*, *J *= 8.0 Hz, H-8), 7.72 (1H, *s*, H-3), 7.53 (1H, *dd*, *J *= 8.3, 1.5 Hz, H-6), 7.27 (1H, *t*, *J *= 8.0 Hz, H-7), 6.47 (1H, *dd*, *J *= 17.1, 10.4 Hz, H-2’), 5.15 (2H, *m*, H-3’), 1.66 (6H, H-4’ , H-5’); ^13^C- NMR (pyridine-*d*_5_, 125 MHz): δ 184.4 (C-9), 153.2 (C-1), 149.7 (C-5), 148.7 (C-2’), 148.6 (C-4b), 144.2 (C-4a), 138.1 (C-4), 128.7 (C-2), 124.7 (C-3), 124.6 (C-7), 123.4 (C-8a), 122.2 (C-6), 116.1 (C-8), 112.3 (C-3’), 110.1 (C-9a), 41.1 (C-1’), 27.2 (C-4’, C-5’); HREIMS [M-H]^-^*m/z* 311,0934, C_18_H_16_O_5_ requires 311.0919.

*Pyrojacareubine* (**3**): Orange-yellow powder; UV (MeOH) λ_max_ (log ε): 329 (3.42), 276 (4,18), 220 (4.08); IR ν_max_ (ns) 3330, 1650, 1580, 1490, 1450, 1290, 1220 cm^−1^; ^1^H-NMR (pyridine-*d*_5_, 500 MHz): δ 13.05 (1H, *s*, 1-OH), 7.46 (1H, *s*, H-8), 6.90 (1H, *d*, *J *= 10.0 Hz, H-2’), 6.45 (1H, *d*, *J *= 10.1 Hz, H-7’), 6.27 (1H, *s*, H-4), 5.74 ( 1H, *d*, *J *= 10.1 Hz, H-8’), 5.62 (1H, *d*, *J *= 10.0 Hz, H-3’), 1.55 (6H, *s*, H-10’, H-11’), 1.50 (6H, *s*, H-5’, H-6’); ^13^C-NMR (pyridine-*d*_5_, 125 MHz): δ 180.8 (C-9), 164.2 (C-1), 161.5 (C-3), 146.4 (C-4a), 131.0 (C-8’), 127.2 (C-3’), 121.4 (C-7’), 118.2 (C-7), 115.2 (C-2’), 113.5 (C-8), 110.4 (C-8a), 103.3 (C-2), 101.6 (C-9a), 99.4 (C-4), 78.9 (C-9’), 78.2 (C-4’), 28.4 ( C-10’, C-11’), 28.3 (C-5’, C-6’); HREIMS [M-H]^-^*m/z* 391.1177, C_23_H_19_O_6_ requires 391.1182. 

*Isogarcinol* (**4**): Brown powder; [*α*]^25^_D_ = −160 (*c* = 1.0, CHCl_3_); UV (MeOH) λ_max_ (log ε): 317 (3.82), 277 (4,14), 233 (4.07); IR ν_max_ (ns) 3290, 2920, 2850, 1730, 1670, 1590, 1520, 1440, 1370, 1290, 1170 cm^-1^; ^1^H- NMR (pyridine-*d*_5_, 500 MHz): δ 8.05 (1H, *d*, *J *= 2.0 Hz, H-12), 7.68 (1H, *dd*, *J *= 8.1, 2.0 Hz, H-16), 7.28 (1H, *d*, *J *= 8.1 Hz, H-15), 5.42 (1H, *tl*, *J *= 6.5 Hz, H-18), 5.09 (2H, *tl*, *J *= 6.5 Hz, H-35, H-25), 3.27 (1H, *dd*, *J *= 13.9, 3.1 Hz, H-29), 3.21 (1H, *ddd*, *J *= 14.4, 10.7, 9.5 Hz, H-24), 2.76 (1H, *dd*, *J *= 13.7, 5.6 Hz, H-17), 2.43 (1H, *dl*, *J *= 14.1 Hz, H-8), 2.42 (1H, *dl*, *J *= 14.1 Hz, H-24), 2.11 (1H, *dd*, *J *= 14.1, 7.3 Hz, H-8), 1.96 (1H, *dl*, *J *= 14.1 Hz, H-34), 1.91 (3H, *s*, H-28), 1.82 (1H, *ddd*, *J *= 14.2, 9.5, 9.5 Hz, H-34), 1.74 (3H, *s*, H-27), 1.71 (3H, *s*, H-21), 1.68 (3H, *s*, H-37), 1.66 (1H, *dt*, *J *= 9.9, 5.0 Hz, H-30), 1.57 (3H, *s*, H-20), 1.56 (3H, *s*, H-38), 1.54 (1H, *m*, H-7), 1.30 (3H, *s*, H-23), 1.23 (3H, *s*, H-32), 1.14 (1H, *dd*, *J *= 13.9, 13.7 Hz, H-29), 1.07 (3H, *s*, H-33), 1.05 (3H, *s*, H-22); ^13^C-NMR (pyridine-*d*_5_, 125 MHz): δ 207.9 (C-9), 195.0 (C-4), 193.0 (C-10), 172.2 (C-3), 171.4 (C-2), 153.7 (C-14), 147.8 (C-13), 134.5 (C-19), 133.7 (C-36), 132.9 (C-26), 130.9 (C-11), 126.4 (C-25), 124.3 (C-16), 122.8 (C-35), 121.7 (C-18), 116.6 (C-12), 116.5 (C-15), 87.2 (C-31), 69.2 (C-5), 52.2 (C-1), 47.0 (C-7), 46.8 (C-6), 43.8 (C-30), 39.8 (C-8), 30.4 (C-34, C-24), 29.4 (C-33), 29.1 (C-29), 27.1 (C-22), 26.7 (C-17), 26.6 (C-20), 26.5 (C-27), 26.2 (C-37), 23.1 (C-23), 21.7 (C-32), 19.0 (C-28), 18.8 (C-21), 18.3 (C-38); HREIMS [M-Na]^+^*m/z* 625.3499, C_38_H_50_O_6_ requires 625.3505.

*7-epi-Isogarcinol* (**5**): Brown powder; [*α*]^25^_D_ = −158 (*c* = 1.0, CHCl_3_); UV (MeOH) λ_max_ (log ε): 319 (3.85), 276 (4,15), 233 (4.12); IR ν_max_ (ns) 3320, 2970, 2930, 1730, 1650, 1590, 1520, 1440, 1370, 1290, 1170 cm^-1^; ^1^H- NMR (pyridine-*d*_5_, 500 MHz): δ 8.13 (1H, *sl*, H-12), 7.72 (1H, *dl*, *J *= 7.7 Hz, H-16), 7.31 ( 1H, *dl*, *J *= 7.6 Hz, H-15), 5.38 (1H, *tl*, *J *= 6.2 Hz, H-18), 5.18 (1H, *tl*, *J *= 6.2 Hz, H-25), 5.13 (1H, *tl*, *J *= 6.6 Hz, H-35), 3.28 (1H, *dd*, *J *= 13.9, 3.2 Hz, H-29), 2.97 (1H, *dd*, *J *= 13.6, 6.4 Hz, H-17), 2.77 (1H, *dd*, *J *= 13.6, 4.8 Hz, H-17), 2.44 (1H, *m*, H-7), 2.43 (1H, *m*, H-8), 2.26 (1H, *dd*, *J * = 14.1, 2.5 Hz, H-24), 1.96 (1H, *m*, H-34), 1.82 (1H, *dd*, *J *= 14.1, 8.5 Hz, H-24), 1.79 (1H, *m*, H-34), 1.77 (1H, *m*, H-8), 1.73 (3H, *s*, H-21), 1.71 (3H, *s*, H-37), 1.65 (1H, *m*, H-30), 1.62 (6H, *s*, H-27, H-28), 1.56 (3H, *s*, H-38)1.55 (3H, *s*, H-20), 1.27 (3H, *s*, H-23), 1.21 (1H, *dd*, *J *= 13.9, 13.7 Hz, H-29), 1.14 (3H, *s*, H-32), 1.08 (3H, *s*, H-33), 0.83 (3H, *s*, H-22); ^13^C-NMR (pyridine-*d*_5_, 125 MHz): δ 207.3 (C-9), 194.9 (C-4), 193.2 (C-10), 170.8 (C-2), 153.7 (C-14), 147.9 (C-13), 134.2 (C-19), 133.7 (C-36), 132.9 (C-26), 131.0 (C-11), 129.1 (C-3), 124.4 (C-16), 123.8 (C-25), 122.9 (C-35), 122.1 (C-18), 117.1 (C-12), 116.5 (C-15), 87.6 (C-31), 71.4 (C-5), 52.2 (C-1), 46.8 (C-6), 43.8 (C-30), 43.1 (C-8), 42.2 (C-7), 30.4 (C-34), 29.2 (C-33), 28.5 (C-24, C-29), 26.2 (C-27), 25.9 ( C-17), 26.2 (C-37, C-20), 22.8 (C-23), 21.7 (C-32), 18.8 (C-21), 18.4 (C-28), 18.3 (C-38), 16.6 (C-22); HREIMS [M-Na]^+^*m/z* 625.3519, C_38_H_50_O_6_ requires 625.3505.

### 3.4. Biological Activities

The extracts and compounds were tested against the chloroquine-resistant FcB1/ Colombia strain of *Plasmodium falciparum* in 96-well plates by measuring [3H]-hypoxanthine incorporation by parasite as previously described [23]. The growth inhibition for each compound concentration was determined by comparing the radioactivity incorporated in the treated culture with that in the control culture maintained on the same plate. The concentrations causing 50% inhibition of parasite growth (IC50) were calculated from the drug concentration-response curves. Chloroquine^®^ was used as a control compound.

The human diploid embryonic lung cells MRC-5 were seeded into 96-well microplates at 2000 cells per well. The cytotoxicity assays were performed according to a published procedure [24]. Taxotere^®^ was used as a control compound.

## 4. Conclusions

A chemical investigation of *Rheedia acuminata* bark was carried out in the framework of a global investigation on French Guiana flora. This study showed that the bark contained a new xanthone, 1,5,6-trihydroxy-3-methoxy-7-geranylxanthone, together with 2-(1’-1’-dimethylprop-2’-enyl)-1,4,5-trihydroxyxanthone. Pyrojacareubin, isogarcinol and 7-*epi*-isogarcinol were isolated from the *Rheedia* genus, for the first time. The two PPAPs isolated from *Rheedia acuminata, *which exhibited cytotoxic and antiplasmodial properties, were likely responsible of the biological activity found in the crude extract. 
